# CTL Responses of High Functional Avidity and Broad Variant Cross-Reactivity Are Associated with HIV Control

**DOI:** 10.1371/journal.pone.0029717

**Published:** 2012-01-04

**Authors:** Beatriz Mothe, Anuska Llano, Javier Ibarrondo, Jennifer Zamarreño, Mattia Schiaulini, Cristina Miranda, Marta Ruiz-Riol, Christoph T. Berger, M. José Herrero, Eduard Palou, Montse Plana, Morgane Rolland, Ashok Khatri, David Heckerman, Florencia Pereyra, Bruce D. Walker, David Weiner, Roger Paredes, Bonaventura Clotet, Barbara K. Felber, George N. Pavlakis, James I. Mullins, Christian Brander

**Affiliations:** 1 IrsiCaixa AIDS Research Institute - HIVACAT, Hospital Germans Trias i Pujol, Badalona, Barcelona, Spain; 2 Lluita contra la Sida' Foundation, Hospital Germans Trias i Pujol, Badalona, Barcelona, Spain; 3 Universitat Autònoma de Barcelona, Barcelona, Spain; 4 Ragon Institute of MGH, MIT and Harvard, Boston, Massachusetts, United States of America; 5 Department of Immunology, LIRAD-Banc de Sang i Teixits, Hospital Germans Trias i Pujol, Badalona, Barcelona, Spain; 6 AIDS Research Group-IDIBAPS, Hospital Clinic, HIVACAT, University of Barcelona, Barcelona, Spain; 7 Department of Microbiology, University of Washington, Seattle, Washington, United States of America; 8 Massachusetts General Hospital, Peptide/Protein Core Facility, Boston, Massachusetts, United States of America; 9 Microsoft Research, Redmond, Washington, United States of America; 10 Howard Hughes Medical Institute, Chevy Chase, Maryland, United States of America; 11 University of Pennsylvania, Philadelphia, Pennsylvania, United States of America; 12 NCI-Frederick, Frederick, Maryland, United States of America; 13 Institució Catalana de Recerca i Estudis Avançats (ICREA), Barcelona, Spain; University of London, St George's, United Kingdom

## Abstract

Cytotoxic T lymphocyte (CTL) responses targeting specific HIV proteins, in particular Gag, have been associated with relative control of viral replication *in vivo*. However, Gag-specific CTL can also be detected in individuals who do not control the virus and it remains thus unclear how Gag-specific CTL may mediate the beneficial effects in some individuals but not in others. Here, we used a 10mer peptide set spanning HIV Gag-p24 to determine immunogen-specific T-cell responses and to assess functional properties including functional avidity and cross-reactivity in 25 HIV-1 controllers and 25 non-controllers without protective HLA class I alleles. Our data challenge the common belief that Gag-specific T cell responses dominate the virus-specific immunity exclusively in HIV-1 controllers as both groups mounted responses of comparable breadths and magnitudes against the p24 sequence. However, responses in controllers reacted to lower antigen concentrations and recognized more epitope variants than responses in non-controllers. These cross-sectional data, largely independent of particular HLA genetics and generated using direct *ex-vivo* samples thus identify T cell responses of high functional avidity and with broad variant reactivity as potential functional immune correlates of relative HIV control.

## Introduction

Several studies in cohorts of clade B and clade C-infected individuals have shown that cytotoxic T-cell (CTL) responses against HIV-1 Gag correlate with relative control of HIV-1 [1], [2], [3], [4]. The rapid re-presentation of epitopes derived from the Gag proteins contained in the infecting viral particles and structural constraints of the Gag protein that complicate CTL escape have been suggested as possible mechanisms that lend Gag-specific CTL responses this superior effectiveness in controlling HIV-1 [5], [6]. However, in all studies reporting beneficial effects of Gag-specific responses, some HIV-1-infected non-controllers mount detectable responses against Gag as well, raising the question as to why these individuals are unable to control their viral replication. A possible answer to this question is that functional characteristics [7], [8], [9], including functional avidity and variant cross-reactivity are distorted in the CTL population in HIV non-controllers. However, some of these characteristics may not be captured reliably when using some standard in vitro antigen test sets and assay systems [10], [11].

In the present study, we analyzed HIV Gag-p24 specific T cell responses in HIV-1 controllers and non-controllers using 18mer and 10 mer peptide sets to compare relative response rates using either longer or shorter test peptides and to determine the functional avidity of these responses as well as their ability to react with naturally occurring sequence variants. Furthermore, the data also allowed to assess whether the most conserved regions within p24 are differentially targeted by HIV-1 controllers and non-controllers in order to provide in vitro relevance for vaccine approaches focusing on such conserved elements (CE) in the viral genome [12], [13].

Although responses to Gag p24 were of comparable breadth and magnitude in HIV-1 controllers and non-controllers when using the 10 mer peptide set, significantly higher avidity responses were seen in controllers, who also showed broader epitope variant cross-reactivity than non-controllers. The data suggest that the maintenance of high avidity responses with broad variant recognition potential is a potential hallmark of controlled HIV-1 infection; a finding that may have important implications in the development of preventative as well as therapeutic vaccine strategies.

## Results

### Gag p24 specific T cell responses in controllers and non-controllers are significantly increased when using 10 mer peptides sets

Chronically HIV-1 infected individuals with controlled HIV infection (n = 25; median viral load 810 RNA copies/ml and median CD4 cell count 642 cells/mm^3^) and non-controlled viral replication (n = 25; viral load median viral load 200,000 RNA copies/ml and median CD4 cell counts 98 cells/mm^3^) were recruited from the HIV Unit in Hospital Germans Trias i Pujol, Badalona, Spain. The study was approved by the Institutional Review Board of the Hospital Germans Trias i Pujol and all individuals provided written informed consent. Median age of individuals was slightly higher for the non-controllers group compared to controllers (44 years-old (24–55) vs 38 years-old (26–56), p = 0.04) but individuals did not significantly differ in time since HIV diagnosis (p = 0.07) (Table 1). The participants were mostly of Caucasian ethnicity (79% Caucasian, 17% Hispanic, 2% African and 2% Asian) and the ethnic origin did not differ between the two groups. HLA diversity was heterogeneous in both groups and individuals expressing HLA-B27, HLA-B57, or HLA-B58 were intentionally excluded from the cohort to avoid bias due to the presence of dominant Gag p24 CTL epitopes restricted by these alleles and to overcome the limitations of past studies in which these alleles were highly over-represented (**Table S1**).

**Table 1:** Demographic and main clinical characteristics of the 25 controllers and 25 non-controllers tested^a^.

	C (n = 25)	NC (n = 25)	P value
Age, years	38 (26.2–55.7)	44.5 (24.3–54.8)	0.04
Time since HIV-1 diagnosis (years)	9.3 (3.5–26.3)	15.9 (1.5–23.3)	0.07
Gender (Female/Male)	F 40%/M 60%	F 40%/M 60%	
HIV risk group			
Heterosexual^b^	6 (24%)	10 (40%)	0.36
Men who have sex with men^b^	8 (32%)	4 (16%)	0.32
Injecting drug users^b^	7 (28%)	9 (36%)	0.76
Other^b^	4 (16%)	2 (8%)	0.66
Last CD4+ T cell counts (cells/mm^3^)	642 (434–1114)	98 (11–361)	<0.001
% CD4 cells	32 (16–50)	9 (1–27)	<0.001
Last HIV-1 RNA levels (copies/ml)	810 (UD^C^-10,000)	200,000 (52,000–1,200,000)	<0.001
HLA alleles representation			
HLA-A (n = 24 alleles)	20 alleles	15 alleles	
HLA-B (n = 34 alleles)	27 alleles	17 alleles	
HLA-C (n = 20 alleles)	17 alleles	15 alleles	

*^a^*Data are expressed as median (min-max range),

*^b^*n, (%),

*^c^*UD: undetectable viremia (<49 copies/ml).

In a first step, the distribution of total HIV-1-specific T-cell responses using a 18 mer overlapping peptide (OLP) set covering the full HIV-1 proteome was assessed in the 50 individuals included. The majority of responses were directed against OLP located in the HIV-1 Gag, Pol and Nef proteins with a relative dominance of Gag/p24 in HIV-1 controllers (p = 0.0336 for Gag, p = 0.0486 for Gag p24), These data confirmed the expected distribution of responses from earlier reports in HIV-1 controllers even though the present cohort was smaller and did not include individuals expressing known protective HLA class I alleles [4], [14]. Of note, the peptide concentrations used were relatively high (14 ug/ml), and in our hands saturating [15], to avoid missing responses due to suboptimal peptide concentrations.

**Figure 1:**
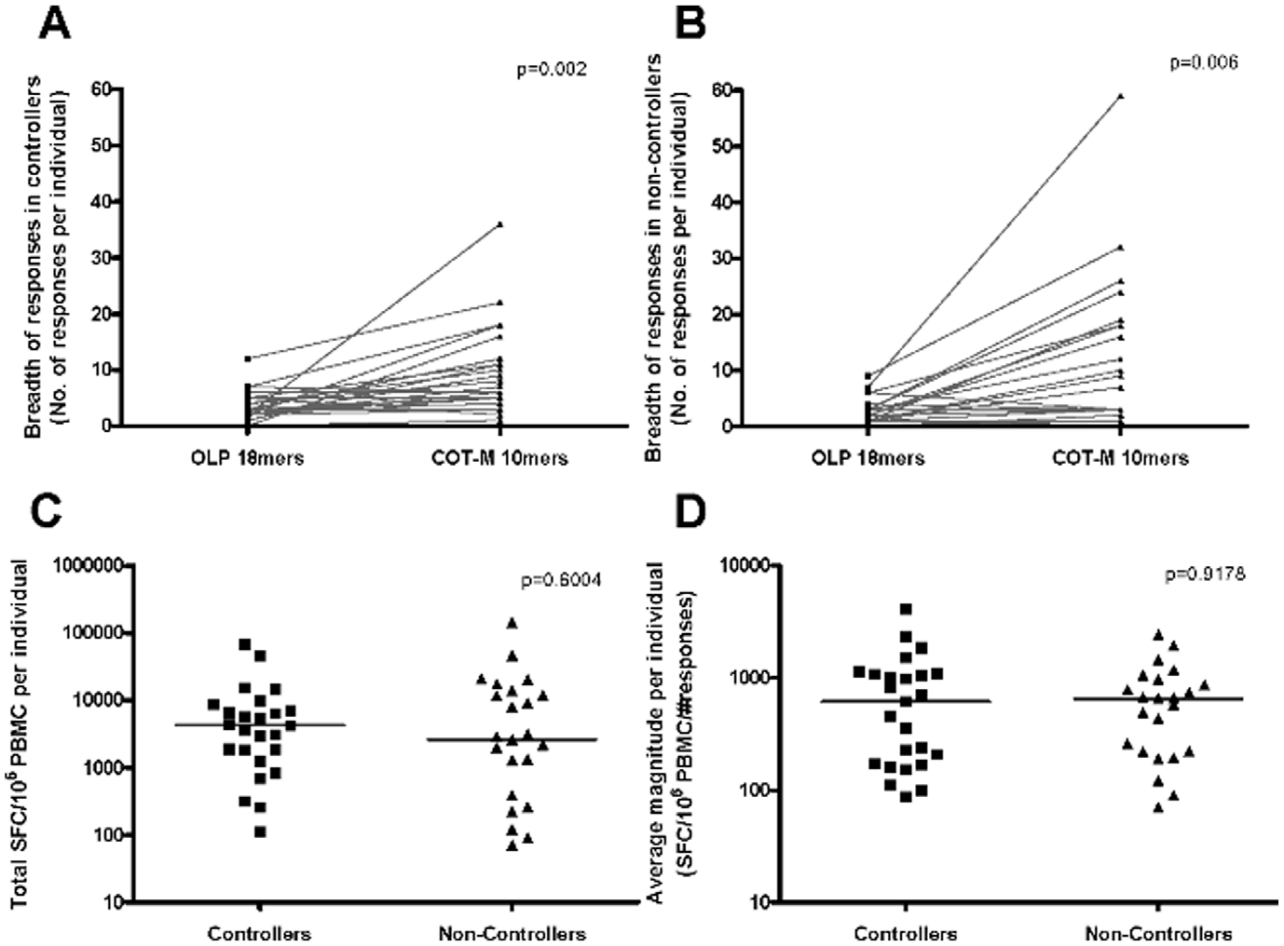
Increased detection of Gag p24 specific responses using a 10 mer peptide set. IFN-γ ELISpot responses against Gag p24 elicited either by consensus B overlapping 18 mer or COT-M 10 mer peptide sets in 25 HIV-1 controllers (**A**) and 25 HIV-1 non-controllers (**B**) P-values reflect the increase in median breadth of responses when using 10 mer peptide sets instead the 18 mer peptides (two-tailed Wilcoxon matched paired test). Total magnitude of responses (**C**) and average magnitude of responses (**D**) to COT-M Gag p24 10 mer peptides are shown for 25 controllers and 25 non-controllers, respectively. Lines represent median values and indicated p values are based on Mann-Whitney t-tests.

To increase the sensitivity of the assay and to discern potential functional differences of Gag responses in non-controllers unable to mediate relative viral control in these subjects, all individuals were tested against a set of 223 10 mer peptides (overlapping by 9 residues) spanning the group M Center-of-Tree (COT-M) Gag p24 sequence (**Figure S1**). Significantly more responses were identified by using the 10 mers in both groups (**Figure 1A,B**; p = 0.0002 for controllers, p = 0.0006 non-controllers). 20 of the 25 individuals in each group showed an increase in the detected responses with the 10 mer test set; while only 3 had equivalent breadth and 2 individuals in each group had one response less compared to the 18 mer peptides. Controllers and non-controllers showed a 2–3-fold increase of their responses which abolished the broader response rates seen in controllers when using the 18 mer peptides (p = 0.4260). Responses detected with the 10 mer peptide set were of comparable magnitude in the two groups, both in terms of total magnitude (median 4,250 vs. 2,600 SFC/10^6^ PBMC in controllers and non-controllers, respectively; p = 0.6004, **Figure 1C**) and the average magnitude of individual responses (median 614 vs. 657 SFC/10^6^PBMC, p = 0.9178, **Figure 1D**). These results demonstrate that Gag p24 specific responses are readily detectable in HIV-1 non-controllers when using a sensitive 10 mer peptide set and that they are unlikely to represent spurious, nonspecific reactivities. The data also show that using 18 mer peptides may potentially miss up to 2/3 of responses, al least in some of the HIV non-controllers and the antigen (i.e. p24) tested here.

### Responses in HIV-1 controllers are of higher functional avidity than in non-controllers and mediate better variant recognition

**Figure 2:**
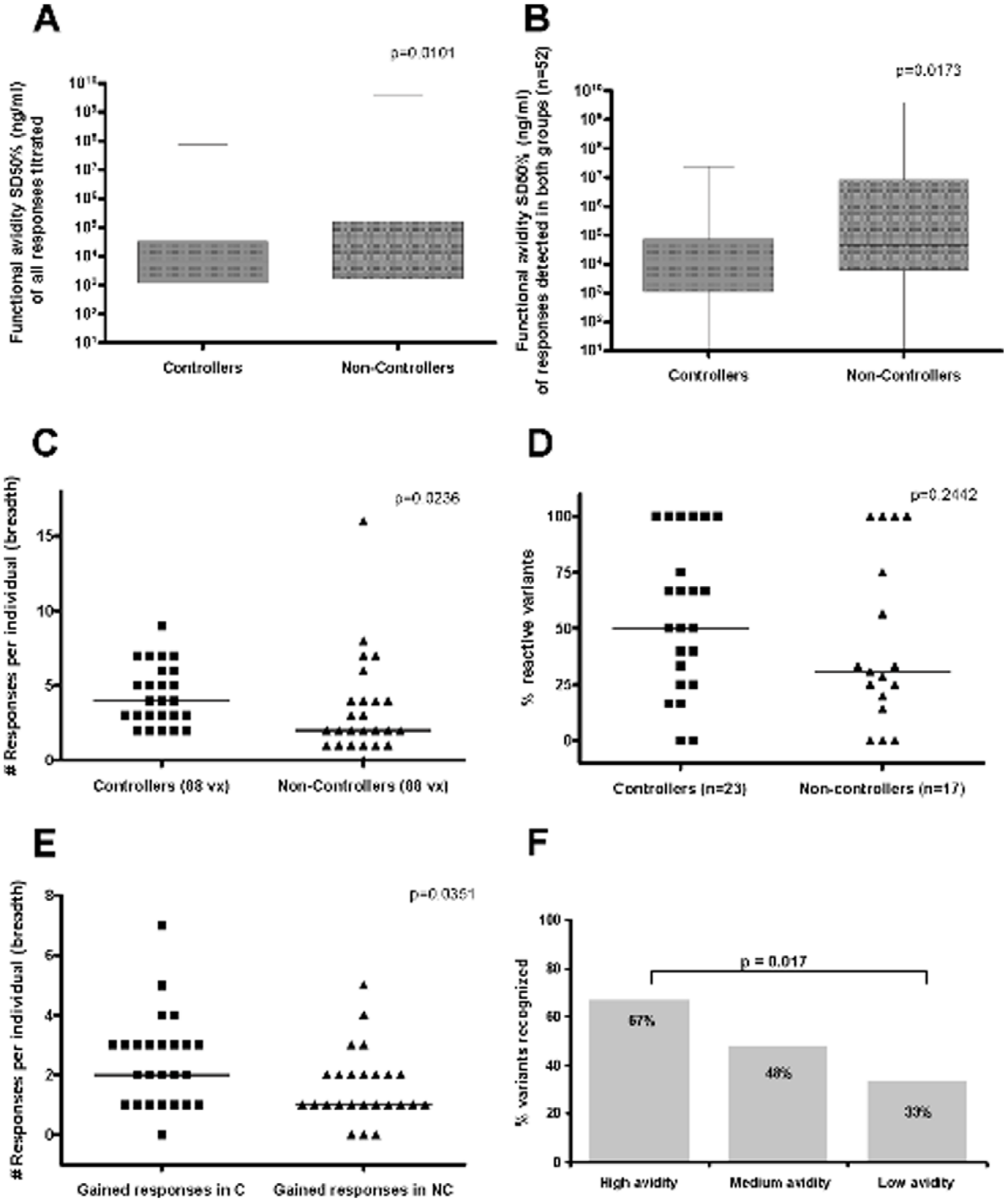
High avidity responses are enriched in HIV-1 controllers and mediate superior variant recognition. (**A**) Comparison of functional avidity of all COT-M Gag p24 responses titrated in controllers (n = 219 responses) vs. non-controllers (n = 255 responses) (**B**) Comparison of functional avidities limited to responses targeting the same 10 mer OLP in the two groups (n = 52 responses, Wilcoxon). In (**C**) the total breadth (number) of the response to the tested COT-M Gag p24 variant peptides (n = 88) is indicated for controllers and non-controllers. (**D**) Shows the percentage of variant peptides that were reactive when the COT-M sequence elicited a response (“cross-reactive responses”) and (**E**) indicates responses to variant peptides for which the COT-M sequence did not elicit a response (“gained responses”). The association between functional avidity and cross-reactivity is shown in (**F**) where responses with functional avidities in either the first quartile of all titrated responses (SD50%<1,401 ng/ml) or the second or third quartile (SD50% 1,401–71,594 ng/ml) or the fourth quartile (SD50%>71,594 ng/ml) were defined as “high”, “intermediate” and low” avidity responses. The percentage of variants that elicited a response was compared between the three groups (Fishers Exact Test).

Data from animal studies and our own analyses in HCV infection suggest that T cell responses of high functional avidity are superior in mediating viral control [3], [16], [17], [18]. We thus tested whether HIV-1 controllers and non-controllers differed in the overall functional avidity of their responses. Based on cell availability, the functional avidity was determined for a total of 474 individual positive responses (219 in controllers and 255 in non-controllers). Controllers indeed showed responses of higher functional avidity (median 6,110 ng/ml, range 0.05–7.6×10^7^) compared to non-controllers (median of 13,548 ng/ml, range 0.64–4×10^9^; p = 0.0101, **Figure 2A**). This difference was more pronounced when the analyses was limited to the 52 10 mer-specific responses that were titrated in both groups (6,998 ng/ml vs. 46,637 ng/ml, respectively; p = 0.0173, **Figure 2B**) While it is possible that even within one 10 mer peptide more than one epitope could be located (i.e. 9 mer optimal epitopes) and that 10 mer responses could be due to presentation of different epitopes on different HLA alleles, the HLA representation between controllers and non-controllers was similar (particularly because HLA-B57, 58, B27 expressing individuals were excluded). Therefore, it is likely that the same epitope in the same HLA context was being targeted in most of the cases included in the matched analysis and that differential allele-effects would not have impacted the comparison between the two groups.

As high avidity responses may be more prone to react with sequence variants in their cognate epitopes, they may provide a crucial advantage in the control of highly variable pathogens such as HIV and HCV [7], [17]. To address whether high avidity responses in HIV controllers would indeed react with more epitope variants, naturally occurring sequence variants were tested for cross-recognition in all 50 subjects using a set of 88 additional 10 mer peptide variants. The median number of responses to these 88 variants was 2-fold greater in controllers (median of 4 responses; range 2–9) than in HIV-1 non-controllers (median 2 responses, range 0–16, p = 0.0236, **Figure 2C**). In particular, controllers showed responses to both the wild-type and variant peptides in half of the cases where a COT-M and a variant peptide was tested (50%) while only 31% in HIV-1 non-controllers reacted to variants (**Figure 2D**). While this did not reach statistical significance, controllers reacted with significantly more variant peptides for which the COT-M sequence did not elicit a response (median of 2 additional responses by inclusion of variants, range 0–7) than the non-controllers (median 1, range 0–5; p = 0.0351; **Figure 2E**). The average magnitude of the variant-specific responses was comparable between controllers (median of 742 SFC/10^6^PBMC, range 90–3,073) and non-controllers (median 473 SFC/10^6^PBMC, range 60–2,707; p = 0.5605, data not shown) indicating that cross-reactive responses in HIV non-controllers were robust, when present.

In order to directly test whether functional avidity was related to the ability to recognize peptide variants, titrated responses were grouped into high, intermediate and low avidity responses and their variant recognition potentials were compared. Indeed, responses with functional avidities in the first quartile of all titrated responses (SD50%<1,401 ng/ml) showed cross-reactivity with their variants in 67% of all cases, whereas fewer (48% and 33%) responses of intermediate or low functional avidity were cross-reactive with their variants, respectively (**Figure 2F**). Collectively, the data demonstrate that high avidity responses were more prevalent in HIV-1 controllers and that these responses mediate superior variant recognition than responses of low functional avidity.

### Conserved regions in Gag p24, containing HLA-B14, -B27 and B57 restricted, protective CTL epitopes are frequently targeted by HIV-1 controllers that do not express protective HLA alleles

**Figure 3:**
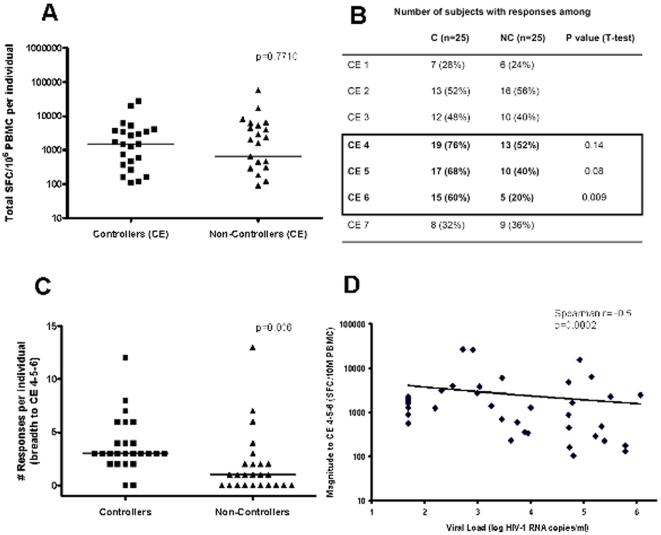
CE containing HLA-B14, -B27 and B57 restricted, protective CTL epitopes are predominantly targeted by HIV-1 controllers. (**A**) The total magnitude of responses to CE regions is compared between HIV controllers and non-controllers. (**B**) The frequency of recognition of the 7 different CE is shown for 25 HIV-1 controllers (C) and 25 non-controllers (NC), respectively. CE regions targeted by at least 50% more controllers than non-controllers are boxed and p-values indicated (T test). (**C**) Breadth of responses to the combination of CE 4+5+6 regions in controllers vs. non-controllers is shown. Horizontal lines represent median values and Mann-Whitney t-test p value is shown. (**D**) Correlation between the cumulative magnitude of responses to CE 4+5+6 and HIV viral loads in all 50 tested individuals is shown (Spearman's rank test).

The high degree of sequence conservation in HIV Gag p24 makes this protein an interesting vaccine component and many vaccine immunogen designs indeed include Gag p24 [12], [19], [20]. A recently developed immunogen sequence is based on a strong focus on the most conserved elements (CE) within Gag p24, excluding variable segments that could contain potential decoy epitopes that may divert the host T cell response towards less valuable targets [13]. These CE were defined as sequence stretches of at least 12 amino acids in length that contain only amino acids residues with at least 98% sequence conservation across all available independent group M sequences [13]. Gag p24 contains 7 of such CE segments, ranging from 12 to 24 amino acids in length and corresponding to a total of 124 residues (**Figure S1**). To validate this immunnogen concept, we stratified the T cell data from the 50 controllers and non-controllers based on the location of the targeted 10 mer, i.e. whether they were located within or outside of these conserved elements. Both groups showed comparable breadth and magnitudes of total CE-specific responses (**Figure 3A**). Also, controllers reacted with significantly more epitope variants located in CE regions than non-controllers (median of 2 responses in controllers vs. 1 response in non-controllers, p = 0.0145, data not shown). Of note, these differences were not due to a suboptimal match between test peptide sequences and autologous viral HIV-1 sequences in the non-controllers as their dominant autologous p24 sequence was in all cases clade B and mostly (99%) represented by the test peptides (**Figure S2**).

Of interest, there were three CE that were recognized by at least 50% more controllers compared to non-controllers (referred to as CE #4, #5, #6), suggesting that these may be preferential targets in HIV-1 controllers (**Figure 3B**). In fact, HIV-1 controllers mounted significantly more responses to at least one of these three CE than did non-controllers (p = 0.0006; **Figure 3C**) and showed a trend towards these being responses of higher functional avidity, even though only a fraction of the overall data points were included in this comparison (median 7,189 ng/ml, range 0.99–2.5×10^7^ vs. median of 17,058 ng/ml, range 16.28–2.62×10^7^; p = 0.0666, data not shown). In addition, the total magnitude of the responses to CE #4+5+6 showed a statistically significant correlation with HIV-1 viral load (r = −0.5, p = 0.0002 by Spearman's rank, **Figure 3D**) across all 50 subjects, suggesting that stronger responses to these three regions may mediate better control of viral replication.

Interestingly, these 3 regions included the well-characterized HLA-B57 restricted TW10 epitope (in CE #4), the HLA-B27 restricted KK10 epitope (in CE #5), and the HLA-B14 restricted DA9 epitope (in CE #6), all of which have been previously associated with containment of in vivo HIV-1 replication [21], [22], [23], [24]. However, as the cohort did not contain any HLA-B57+ and -B27+ individuals and only 2 subjects expressed HLA-B14, the data indicate that mounting responses to these regions is effective even if these protective responses are being restricted by HLA class I molecules different from the originally described restricting HLA molecules [25], [26]. Indeed, the HLA class I allele representation of CE #4, #5 or #6 responders was heterogeneous and not limited to individuals with a few shared HLA alleles (**Table S1**) indicating that the CE regions represent a very rich set of epitopes that are not being blocked from presentation in natural chronic infection and that are able to be recognized in a wide HLA class I context.

## Discussion

Together, our data strongly suggest that the presence of responses of high functional avidity and with broad variant recognition ability is a potential hallmark of controlled HIV-1 infection. To the best of our knowledge, this is the first demonstration of a direct link between high avidity T cell responses, broad variant recognition and in vivo HIV-1 control using ex-vivo blood samples from a cohort with largely unbiased HLA genotypes. Our data also support that standard approaches using 15–20 mer overlapping peptides underestimate the breadth of responses to HIV Gag p24 significantly. While similar findings have been reported in earlier studies, none has addressed this in a systematic way and including a comparably extensive avidity determination as in the present study. More importantly though, since HIV non-controllers profited the most from using a more sensitive peptide set, the data have also important implication for our understanding of HIV immunopathogenesis and vaccine immunogen design: It is not that HIV-1 non-controllers would not mount Gag-specific T-cell responses; rather, they may have either induced low-avidity responses during acute infection or induced originally high functional avidity responses that were lost over the course of HIV-1 infection. This latter interpretation would be in line with results from longitudinal analyses in individuals followed from acute infection time points [27], [28]. Although these analyses were based on fewer individuals and included many donors expressing HLA-B27 and -B57 alleles, clonal exhaustion of high avidity cells in the course of chronic HIV replication is certainly a possible explanation why the HIV non-controllers in our study showed responses of reduced functional avidity. On the other hand, it is interesting to note that in a recent report by Berger et al, high avidity responses were not only not restored upon HAART initiation but were actually further diminished [7], suggesting that possibly other factors than duration and extend of antigenimia impact the measurable avidity of an epitope-specific T cell population.

The wide spread and overlap in the avidity measurements between responses among the two groups is probably the biggest challenge for this kind of study to conclusively demonstrate its potential biological significance. Quite likely, inter-epitope and inter-individual differences hamper a clearer observation. In addition, given that different effector functions are subject to variable activation thresholds [29], the inclusion of additional in vitro read-outs could possibly provide a larger discrimination in the minimal antigen amounts required for responses in HIV controllers and non-controllers. Indeed, as limited effector functions have been previously described for HIV-1 non-controllers, one would expect additional reactivities to occur preferentially in the controllers group. Despite the limitation of assessing a single effector function (IFNγ release) though, our findings are supported by a number of previous studies in animal models and in HIV and HCV infection that have assessed the relationship between virus control/clearance, functional avidity and variant recognition [17], [30], [31], [32], [33], [34], [35], [36], [37]. Moreover, none of earlier studies in humans has been based on the extensive number responses analyzed here (close to 500 titrated responses) and most used either *in vitro* expanded short-term T cell lines or T cell clones [17], [32]. While analyzing the functional avidity in clonal T cell populations allows eliminating some of the inherent heterogeneity faced in cross-sectional, directly ex-vivo studies, the *in vitro* selection and expansion of epitope-specific clones may be biasing results too, as shown for HCV specific responses where short-term culture consistently increased the functional avidity compared to directly ex-vivo isolated cells [17]. On the other hand, our data may be limited by the use of 10 mers instead of optimal epitopes for the determination of functional avidity. However, many optimally defined epitopes in Gag are 10 mers and, it has frequently been shown that 9 and 10 mers can have similar SD50%. In fact, in many cases, the definition of optimal epitopes is more driven by the shorter length of 9 mers rather than a substantially lower SD50% [26], suggesting that the 10 mer approach used here is an acceptable approximation to avoid biases or missing responses by alternative optimal epitope or 9-mer approaches.

In order to control for some of the heterogeneity in our data set, we also compared SD50% between matched responses targeting the same 10 mer peptide in the controller and non-controller group. This indeed enhanced the otherwise modest differences in SD50% between the two groups considerably and was statistically significant despite the much smaller number of responses analyzed. The limited difference in SD50% for the overall analysis may also be due to variable activation thresholds for specific CTL effector functions [29]. As such, the observed difference in the SD50% necessary for IFNγ release may not be directly relevant for the improved viral control in vivo but may still reflect a more avid and thus more effective interaction between the CTL and the antigen-presenting cell, regardless of the ensuing cascade of effector function(s).

As mentioned above, high functional avidity may also render CTL more prone to immune senescence or clonal exhaustion, particularly in individuals with suboptimal control of viral replication. The data from cleared and chronic HCV infection support this idea as only HCV clearers seem to maintain responses of high functional avidity in the absence of possible sources of residual antigen [17]. Thus, while high avidity responses may win out during the induction phase of the virus-specific T cell response, these cells may be preferentially lost over time if viral antigenemia cannot be controlled sufficiently well [28], [38]. Whether such losses of high avidity responses correspond to changes in the clonal composition of the CD8 T cell response or to a gradual decrease in their functional avidity due to altered cell reactivity/signaling needs to be further addressed in different clinical settings, as the existing data discussing the ‘cause/effect’ quandary are conflicting and generally limited to responses restricted by few selected HLA alleles [28], [32], [39], [40], [41], [42].

Further studies will ideally also include other highly conserved regions in the viral genome outside Gag p24, which may serve as additional components of vaccine immunogens. Such extended analyses would also increase the number of responses per individual, which in the present study is relatively small given that only a short segment of the entire viral proteome was analyzed. Despite this focus on p24, our comparisons reached statistical significance and compared well to the breadth of responses reported in earlier studies looking at responses to the entire HIV proteome or optimally defined HIV CTL epitopes [4], [14], [15], [25]. Together with the results here, these earlier analyses also provide support that the detected responses are generally CD8 T-cell mediated, particularly when testing short 10 mer peptides and that they are HIV-specific since testing with even optimally defined short HIV-derived CTL epitopes did not readily elicit responses in HIV negative individuals [15], [25].

Given these considerations, our study suggests that HIV-1 controllers mount ex-vivo responses of significantly higher functional avidity than HIV-1 non-controllers. Since the high avidity responses were also more apt to react with epitope variants, their induction by a future HIV-1 vaccine may be crucial to prevent rapid viral escape from the vaccine induced immune response. Finally, as the data presented here confirm findings in HCV infection, they strongly suggest that the ability to maintain T cell responses of high functional avidity is a more general hallmark of effective immune control of infections with highly variable pathogens.

## Materials and Methods

### Synthetic peptides set

An overlapping peptide set of 223 peptides of 10 amino acids in length (overlapping by 9 residues) spanning the entire group M Center-of-Tree (COT-M) Gag p24 sequence was synthesized using 9-Fluorenylmethyloxycarbonyl (Fmoc)- chemistry. Additional 88 10-mer peptides were generated to cover the most frequently occurring variants in the 7 most conserved (CE) regions. We also included a previously described overlapping peptide (410 18 mers OLP) set spanning the entire viral proteome [4], [14] based on the 2001 consensus-B sequence (http://hiv-web.lanl.gov/content/hiv-db/CONSENSUS/M_GROUP/Consensus.html). Peptides were 18 mers varying from 15–20 amino acids in length and overlapping by 10 amino acids, designed using the PeptGen algorithm at the Los Alamos HIV database (http://www.hiv.lanl.gov/content/sequence/PEPTGEN/peptgen.html).

### IFN-γ ELISpot assay

PBMC were separated from whole blood within 4 h of venopuncture and used directly for the IFN-γ ELISpot. Each COT-M Gag p24 peptide was tested individually and added at a final concentration of 14 µg/ml. For all assays, between 75,000–100,000 PBMC per well were added in 140 ul of R10 96-well polyvinylidene plates (Millipore, Bedford, MA). The IFN-γ Mabtech kit was used following manufacturer instructions. In parallel, CTL responses to the clade B full proteome were assessed using the 18 mer peptide set in a previously described optimized peptide matrix, followed by deconvolution of reactive pools and reconfirmation of each response at a single peptide level on the following day and tested at the same concentration of 14 µg/ml [14]. The number of spots was counted using a “CTL ELISpot Reader Unit” and the magnitude of responses was expressed as spot forming cells (SFC) per million input cells. The threshold for positive responses was defined as at least 5 spots per well and responses exceeding the “mean number of spots in negative control wells plus 3 standard deviations of the negative control wells” and “three times the mean of negative control wells”, whichever was higher. As a conservative approach and not to overestimate the breadth of responses, positive responses to 3 consecutive 10 mers in the COT-M Gag p24 peptide set were counted as 1 unique response. Similarly, reactivity to 2 consecutive 18 mer OLP was counted as 1 response. The highest magnitude of the sequential responses was taken as the magnitude for each identified response.

### Determination of functional avidity

The functional avidity of responses was determined by performing serial 10-fold limiting peptide dilutions ranging from 100 µg/ml to 10 pg/ml using the 10 mer peptide set; in duplicate whenever enough PBMC were available. Half-maximal stimulatory antigen doses (SD50%) were calculated as the peptide concentration needed to achieve a half-maximal number of spots in the ELISpot assay calculated by a sigmoidal dose response curve fit using GraphPad Prism4.

### Gag p24 sequencing

Viral RNA was extracted from 1 millilitre of plasma spun at 25000 rpm for 1 hour (QIAamp Viral RNA Kit™, QIAGEN, Valencia, CA). The whole Gag region was reverse-transcribed and amplified in a One-Step reaction (SuperScript® III One-Step RT-PCR System with Platinum® Taq High Fidelity, Invitrogen, Carlsbad, CA) under the following conditions: 30 min at 52°C for the reverse transcription step; 2 min at 94°C; followed by 35 cycles at 94°C during 30 sec, 58°C during 30 sec and 68°C during 2 min; followed by a final extension step at 68°C during 5 min. Primers used for the RT-PCR were: Gag U761 (HXB2: 761–778) 5′-TTT GAC TAG CGG AGG CTA G-3′ and Gag D2397 (HXB2: 2397–2376) 5′-CCC CTA TCA TTT TTG GTT TCC A-3′. One microliter of the RT-PCR product was subsequently used as a template for a nested PCR (Platinum® Taq DNA Polymerase High Fidelity, Invitrogen, Carlsbad, CA), using primers p24 U1070 (HXB2: 1070–1088) 5′-TAA AAG ACA CCA AGG AAG CT and p24 D2063 (HXB2: 2063–2044) 5′-TCT TTC ATT TGG TGT CCT TC-3′. PCR cycling conditions were: 2 min at 94°C; followed by 35 cycles at 94°C during 30 sec, 54°C during 30 sec and 68°C during 2 min; followed by a final extension step at 68°C during 5 min. The final PCR products were column-purified (QIAquick PCR Purification Kit, QIAGEN, Valencia, CA) and sequenced bidirectionally. Sequences were assembled using Sequencher® 4.10.1 (Genecodes Corp. MI). Assembled sequences were codon-aligned using the Hidden Markov Model implemented in the HIValign tool (www.lanl.hiv.gov). Autologous Gag p24 bulk sequences were obtained for 22 of the 25 HIV-1 non-controllers included in our study. Sequences were submitted to Genbank; accession numbers BCN-NC-1.sqn BCN-NC-1 JQ246370-246391.

### Statistical analyses

All values are presented as median values unless otherwise stated. GraphPad Prism version 4.0 for Windows (San Diego, CA) was used to compare response rates in both groups and subgroup analyses. Mann-Whitney test and Wilcoxon matched paired test were used for unpaired and paired comparisons, respectively. Spearman rank correlation was used to assess association.

## Supporting Information

Figure S1 **COT-M Gag-p24 sequence and location of CE segments.** The Center-of-tree (COT) M sequence is indicated for entire Gag p24. The location of known optimally-defined CTL epitopes listed at the Los Alamos HIV database, are indicated above the protein sequence while the shaded boxes beneath indicate the 7 CE segments and variant (down) residues included in this study.
(TIF)

Figure S2 **Autologous Gag-p24 CE sequences in 21 HIV-1 non-controllers.** Shaded boxes indicate the 7 CE sequences located within in p24 with variant residues included (separated by “/”. The amino acid sequences of autologous Gag p24 bulk sequences obtained from 22 HIV non-controllers are shown.
(TIF)

Table S1 **HLA genotypes of the 25 controllers and 25 non-controllers tested.**
(DOC)
