# Supplementary figures and images for: CTL Responses of High Functional Avidity and Broad Variant Cross-Reactivity Are Associated with HIV Control
Source: PLoS One. 2012 Jan 4;7(1):e29717. doi: 10.1371/journal.pone.0029717 (PMC3251596; doi:10.1371/journal.pone.0029717)

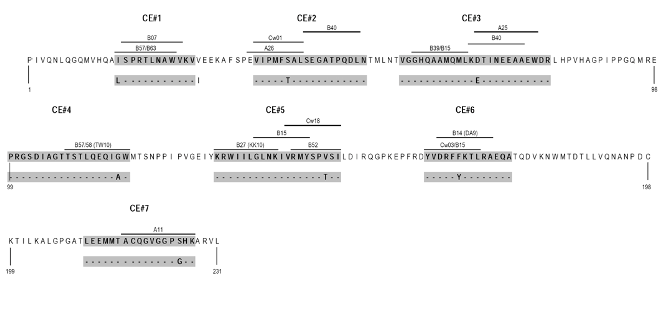

Supplement: Figure S1 — COT-M Gag-p24 sequence and location of CE segments. The Center-of-tree (COT) M sequence is indicated for entire Gag p24. The location of known optimally-defined CTL epitopes listed at the Los Alamos HIV database, are indicated above the protein sequence while the shaded boxes beneath indicate the 7 CE segments and variant (down) residues included in this study. (TIF) [file pone.0029717.s001.tif]

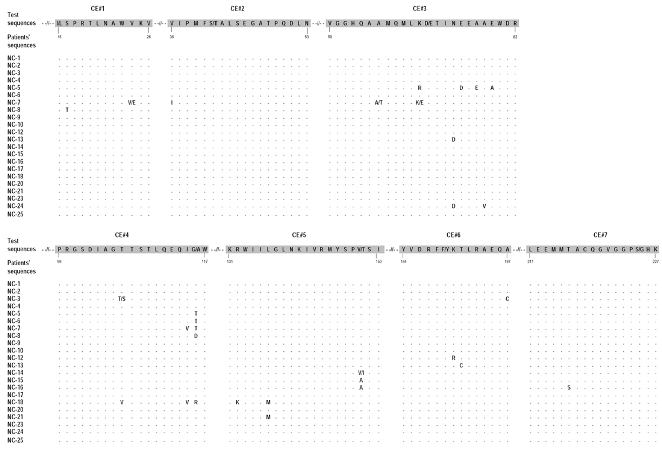

Supplement: Figure S2 — Autologous Gag-p24 CE sequences in 21 HIV-1 non-controllers. Shaded boxes indicate the 7 CE sequences located within in p24 with variant residues included (separated by “/”. The amino acid sequences of autologous Gag p24 bulk sequences obtained from 22 HIV non-controllers are shown. (TIF) [file pone.0029717.s002.tif]
