# Supplementary material for: CTL Responses of High Functional Avidity and Broad Variant Cross-Reactivity Are Associated with HIV Control
Source: PLoS One. 2012 Jan 4;7(1):e29717. doi: 10.1371/journal.pone.0029717 (PMC3251596; doi:10.1371/journal.pone.0029717)
Supplement: Table S1 — HLA genotypes of the 25 controllers and 25 non-controllers tested. (DOC) [file pone.0029717.s003.doc]

**Table S1**. HLA genotypes of the 25 controllers and 25 non-controllers tested

| **Gates ID** | **HLA-A1** | **HLA-A2** | **HLA-B1** | **HLA-B2** | **HLA-C1** | **HLA-C2** |
| --- | --- | --- | --- | --- | --- | --- |
| **C-1** | 3001 | 3601 | 4201 | 4201 | 17MN | 17MN |
| **C-2** | 0201 | 0205 | 0702 | 1402 | 0702 | 0802 |
| **C-3** | 0101 | 3001 | 1302 | 3503 | 0602 | 1203 |
| **C-4** | 0205 | 3201 | 5001 | 5201 | 0602 | 1202 |
| **C-5** | 1101 | 2402 | 4403 | 5201 | 1202 | 1601 |
| **C-6** | 2402 | 3201 | 3503 | 4002 | 202 | 1203 |
| **C-7** | 3002 | 6802 | 1501 | 5301 | 0202 | 0401 |
| **C-8** | 0201 | 2402 | 1401 | 1402 | 0802 | 0802 |
| **C-9** | 0201 | 3301 | 1402 | 3801 | 0802 | 1203 |
| **C-10** | 0101 | 2402 | 1517 | 5101 | 0102 | 0701 |
| **C-11** | 0201 | 0201 | 1402 | 3924 | 0701 | 0802 |
| **C-12** | 1101 | 2402 | 3501 | 5201 | 0401 | 1202 |
| **C-13** | 0101 | 6802 | 1402 | 3502 | 0401 | 0802 |
| **C-14** | 0101 | 6901 | 0801 | 5501 | 0102 | 0701 |
| **C-15** | 0202 | 3002 | 1516 | 4403 | 0401 | 1402 |
| **C-16** | 3201 | 3303 | 1510 | 4002 | 0202 | 0304 |
| **C-17** | 2402 | 6601 | 1801 | 5107 | 0701 | 1402 |
| **C-18** | 0201 | 6801 | 1501 | 5101 | 0304 | 1502 |
| **C-19** | 1101 | 3303 | 3501 | 5301 | 0401 | 0401 |
| **C-20** | 0201 | 3201 | 1302 | 1501 | 0303 | 0602 |
| **C-21** | 0211 | 2601 | 4402 | 5202 | 1502 | 1502 |
| **C-22** | 0301 | 1101 | 0702 | 5201 | 0702 | 1202 |
| **C-23** | 1101 | 2402 | 0702 | 5101 | 0702 | 1502 |
| **C-24** | 0301 | 1101 | 1301 | 5501 | 0102 | 0403 |
| **C-25** | 0201 | 2501 | 3901 | 4402 | 0501 | 1203 |
| **NC-1** | 0201 | 0301 | 0702 | 1801 | 0702 | 1205 |
| **NC-2** | 0201 | 0301 | 1801 | 3501 | 0401 | 0701 |
| **NC-3** | 0201 | 0201 | 3502 | 4101 | 0401 | 17MN |
| **NC-4** | 2402 | 2402 | 4501 | 5101 | 1402 | 1601 |
| **NC-5** | 2301 | 2402 | 4402 | 4405 | 0202 | 0501 |
| **NC-6** | 2902 | 6801 | 1801 | 4501 | 0501 | 0602 |
| **NC-7** | 1101 | 2601 | 0801 | 4403 | 0202 | 0701 |
| **NC-8** | 0101 | 2601 | 3503 | 3701 | 0602 | 1203 |
| **NC-9** | 2601 | 2902 | 3801 | 4403 | 1203 | 1601 |
| **NC-10** | 1101 | 3002 | 1801 | 3503 | 0401 | 0501 |
| **NC-11** | 0201 | 0205 | 1801 | 4402 | 0202 | 0501 |
| **NC-12** | 2601 | 3201 | 1401 | 3503 | 0401 | 0802 |
| **NC-13** | 3201 | 74AB | 1401 | 5001 | 0602 | 0802 |
| **NC-14** | 0201 | 2902 | 3503 | 4403 | 1203 | 1601 |
| **NC-15** | 0201 | 0201 | 1503 | 4402 | 0202 | 0501 |
| **NC-16** | 0301 | 2501 | 1801 | 3801 | 1203 | 1203 |
| **NC-17** | 0101 | 0201 | 1801 | 4403 | 0701 | 1601 |
| **NC-18** | 0205 | 3002 | 1801 | 3502 | 0401 | 0501 |
| **NC-19** | 0201 | 0301 | 0702 | 4402 | 0501 | 0702 |
| **NC-20** | 0201 | 0301 | 0702 | 3801 | 1203 | 1203 |
| **NC-21** | 0201 | 0301 | 0702 | 1801 | 0501 | 0702 |
| **NC-22** | 0201 | 2402 | 1801 | 3701 | 0501 | 0602 |
| **NC-23** | 2301 | 2402 | 1503 | 3543 | 0102 | 0210 |
| **NC-24** | 0201 | 2902 | 0702 | 4403 | 0702 | 1601 |
| **NC-25** | 2402 | 2901 | 3503 | 3503 | 0401 | 0401 |
